# High Antioxidant Activity Facilitates Maintenance of Cell Division in Leaves of Drought Tolerant Maize Hybrids

**DOI:** 10.3389/fpls.2017.00084

**Published:** 2017-02-02

**Authors:** Viktoriya Avramova, Hamada AbdElgawad, Ivanina Vasileva, Alexandra S. Petrova, Anna Holek, Joachim Mariën, Han Asard, Gerrit T. S. Beemster

**Affiliations:** ^1^Research Group of Integrated Molecular Plant Physiology Research, Department of Biology, University of AntwerpAntwerp, Belgium; ^2^Department of Botany, Faculty of Science, University of Beni-SuefBeni-Suef, Egypt

**Keywords:** maize, drought tolerance, leaf meristem, redox regulation, oxidative stress, kinematic analysis, leaf growth, enzyme activity

## Abstract

We studied the impact of drought on growth regulation in leaves of 13 maize varieties with different drought sensitivity and geographic origins (Western Europe, Egypt, South Africa) and the inbred line B73. Combining kinematic analysis of the maize leaf growth zone with biochemical measurements at a high spatial resolution allowed us to examine the correlation between the regulation of the cellular processes cell division and elongation, and the molecular redox-regulation in response to drought. Moreover, we demonstrated differences in the response of the maize lines to mild and severe levels of water deficit. Kinematic analysis indicated that drought tolerant lines experienced less impact on leaf elongation rate due to a smaller reduction of cell production, which, in turn, was due to a smaller decrease of meristem size and number of cells in the leaf meristem. Clear differences in growth responses between the groups of lines with different geographic origin were observed in response to drought. The difference in drought tolerance between the Egyptian hybrids was significantly larger than between the European and South-African hybrids. Through biochemical analyses, we investigated whether antioxidant activity in the growth zone, contributes to the drought sensitivity differences. We used a hierarchical clustering to visualize the patterns of lipid peroxidation, H_2_O_2_ and antioxidant concentrations, and enzyme activities throughout the growth zone, in response to stress. The results showed that the lines with different geographic region used different molecular strategies to cope with the stress, with the Egyptian hybrids responding more at the metabolite level and African and the European hybrids at the enzyme level. However, drought tolerance correlated with both, higher antioxidant levels throughout the growth zone and higher activities of the redox-regulating enzymes CAT, POX, APX, and GR specifically in leaf meristems. These findings provide evidence for a link between antioxidant regulation in the leaf meristem, cell division, and drought tolerance.

## Introduction

Drought is one of the most important environmental factors that adversely affects plant growth, reducing yield quality and quantity of economically important crops throughout the world (Boyer, 1982; Tollenaar and Lee, 2002). Since drought is predicted to become an increasing problem in future climate conditions (Burke et al., 2009; Lobell et al., 2011; IPCC, 2014), an important challenge for plant biologists and breeders is to improve drought tolerance of crops. This is a difficult task due to the diverse strategies adopted by the plants to escape, avoid, or tolerate drought, and the dependence of the response on the timing and severity of the stress and the plant organ affected (Nguyen et al., 2004). Nevertheless, big differences in drought sensitivity between crop varieties depending on contrasting adaptive and survival strategies have been identified (Basu et al., 2010), but the physiological basis of these differences are still poorly understood.

Recently, we studied the effect of drought on the growth of the inbred line B73 and 8 commercial hybrids with contrasting drought tolerance from Europe and South Africa. By linking non-invasive whole-plant phenotyping to detailed kinematic studies, we showed differences in adaptation strategies between lines from different regions and with different drought sensitivity in root and shoot growth, but also meristem size, number of cells in the meristem and size of the growth zone of the leaves under drought stress conditions. We demonstrated that growth of drought tolerant hybrids was less reduced, due to differences in developmental rate, shoot growth rate, photosynthesis, and root system architecture (Avramova et al., 2016).

Prolonged or severe water limitation, like several other abiotic and biotic stressors, causes drastic changes in ion and water homeostasis and results in oxidative stress through accumulation of reactive oxygen species (ROS; Fridovich, 1995; Bolwell et al., 2002; Shigeoka et al., 2002; Cruz de Carvalho, 2008). Changes in ROS not only affect the stability and functioning of macromolecules, it also alters redox-mediated cell signaling. For example, different ROS are involved in the regulation of leaf expansion (Schmidt et al., 2016) and can play a dual role in the regulation of cell proliferation and cell expansion, depending on their type and amount (Schopfer, 1996; Liszkay et al., 2004; Tsukagoshi et al., 2010). Therefore, ROS levels are tightly controlled by antioxidant mechanisms involving enzymes [e.g., catalases (CAT), superoxide dismutases (SOD), peroxidases (POX)], components of the ascorbate-glutathione (Foyer-Halliwell-Asada) cycle, and small molecular antioxidants (e.g., carotenoids, polyphenols, anthocyanins, tocopherols; Rodriguez et al., 2002; Apel and Hirt, 2004). Additional compounds, such as osmolytes, proteins, and sugars, may also contribute to ROS scavenging (Xiong and Zhu, 2002).

Because of their function in controlling the cellular redox status, all these enzymes and metabolites can play a role in the protection against drought stress and provide a mechanism for improved drought tolerance. To evaluate this possibility, we compared antioxidant levels and antioxidant enzyme activities in the growing tissues of lines with different sensitivity to drought. To this end we used the maize leaf, because it provides an excellent experimental model for molecular studies of the developing tissues (Avramova et al., 2015b). After emergence, the leaf enters a period of several days with steady-state growth, which allows spatial quantification of cell division and expansion rates by kinematic analysis (Fiorani and Beemster, 2006). The size of its growth zone (ca. 10 cm starting from the leaf base) allows sampling for molecular and biochemical analyses at sub-zonal resolution (Nelissen et al., 2012). This provides the opportunity to conduct a high resolution examination of the molecular regulation of cell division and cell expansion under optimal and suboptimal conditions such as drought stress. Because of technical reasons, such resolution cannot be achieved in Arabidopsis, (Avramova et al., 2015b). With this approach, we have shown the crucial role of the leaf meristem size control by gibberellic acid in determining leaf elongation rates under control conditions (Nelissen et al., 2012). In roots, the use of kinematic analysis revealed that drought stress inhibited cell expansion in the elongation zone, but not the root tip where cell division occurs (Sharp et al., 2004). This could be related to increased apoplastic antioxidant enzyme levels in the apical region (Zhu et al., 2007) and elevated H_2_O_2_ levels (Voothuluru and Sharp, 2013), suggesting that the apical (meristematic) region may be protected from ROS to maintain growth under drought conditions.

Here, we investigated the potential involvement of ROS regulation in contrasting drought tolerance between the lines from Western Europe and South Africa, that we phenotyped previously (Avramova et al., 2016), supplemented with Egyptian lines with contrasting salt tolerance to further increase the genetic diversity. We compared the cellular basis of the growth response to drought conditions determined by means of kinematic analysis with antioxidant metabolite concentrations and enzyme activities along the growth zone. Moreover, we tested mild and severe stress conditions, to understand potential differences in the underlying molecular responses. Mild drought effects are not often investigated, despite the importance of this condition in crop yields.

## Materials and methods

### Maize lines

Fourteen maize (*Zea mays*) lines were used as a basis for our studies: The inbred line B73 (Iowa Stiff Stalk Synthetic), four hybrid lines from Western Europe: PR39D23 (EU1), P7345 (EU2), PR39T83 (EU3), PR39F58 (EU4), four from South Africa: 33H56 (AF1), 33Y74 (AF2), 3442 (AF3), 31MO9 (AF4), and five from Egypt: SC 128 (EG1), SC 131 (EG2), SC 130 (EG3), SC 161 (EG4), SC 167(EG). Seeds from the Western-European and the South-African hybrid maize lines were generously provided by DuPont Pioneer and seeds from the Egyptian lines were provided by Sids Research Station, Agricultural Research Centre, Beni-Suef, Egypt. Based on field trait evaluation, four hybrid lines were rated as drought tolerant (tEU1, tEU4, tAF1, tAF3, for more detailed information see Avramova et al., 2016), and two were rated as salt-tolerant (tEG1 and tEG2).

### Growth experiment

Maize seedlings were grown in a growth chamber under controlled conditions [16 h day/8 h night, 25°C/18°C day/night (d/n), 300–400 μE.m^−2^ s^−1^ Photosynthetically Active Radiation, provided by high pressure sodium lamps]. For control plants the pots were re-watered daily to maintain a Soil Water Content (SWC) of 54%. For drought treatments water contents were allowed to drop after sowing to 43% SWC (mild stress, no wilting), and 34% SWC (severe stress, leaves are wilting during the day), respectively, where they were maintained. Three days after emergence of the 5th leaf, the plants were harvested and the growth zone (the first 10 cm from the leaf basis) of leaf five of each plant was cut in ten segments of 1 cm and the samples were stored at −80°C for further measurements (ROS and antioxidant quantification, enzyme activities).

### Kinematic analysis

The kinematic analysis was done according to an established protocol (Rymen et al., 2010). It entails leaf-elongation rate and final leaf length measurements, measurements of the cell-length profile along the axis of the leaf, and estimation of the size of the leaf basal meristem by locating mitotic cells in DAPI-stained leaf sections. The details of the protocol are described by Avramova et al. (2016). At least 5 plants were measured per line/treatment combination.

### Biochemical measurements

Each measurement was done on 1-cm segments of the 10 cm leaf growth zone. Three biological replicates (each consisting of 2–3 pooled plants) were measured per line.

#### Determination of H_2_O_2_

For H_2_O_2_ determination, 100 mg of the samples were homogenized in 1 ml of 5% TCA (Velikova et al., 2000), by using a MagNALyser (Roche, Vilvoorde, Belgium). Homogenates were centrifuged (14000 rpm, 30 min) and xylenol orange dye reagent (Bellincampi et al., 2000) was added to supernatant. After 45 min incubation, the Fe^3+^-xylenol orange complex was measured at 595 nm.

#### Determination of malondialdehyde (MDA)

MDA was extracted in 2 ml 80% ethanol and measured by using a thiobarbituric acid-malondialdehyde (TBA-MDA) assay (Hodges et al., 1999). The quantity of MDA (μmol) was calculated by the formula: ([6.45 × (A532–A600)–0.56 × A440]/0.478).

#### Antioxidant capacity

The Ferric Reducing Ability of Plasma (FRAP) assay was used to estimate the antioxidant capacity of plant extracts (Benzie and Strain, 1996). Extracts of plant tissue were prepared in 80% (v/v) ethanol and were mixed with 0.3 M acetate buffer (pH 3.6), containing 10 mM 2,4,6 Tris (2 pyridyl) s-triazine (TPTZ) and 200 mM FeCl_3_. The absorbance was measured at 600 nm in a microplate reader. 6-Hydroxy-2,5,7,8-tetramethylchroman-2-carboxylic acid (Trolox) was used as a standard.

#### Polyphenol concentration

Plant tissue extracts were prepared in 80% (v/v) ethanol. Polyphenol concentration was determined by using Folin–Ciocalteu reagent (Gálvez et al., 2005). Absorbance was measured at 765 nm. Gallic acid was used as a standard.

#### Flavonoid concentration

Estimation of total flavonoid content was done by preparing plant tissue extracts in reaction buffer, containing 10% aluminum chloride and 1 M potassium acetate (Chang et al., 2002). After 30 min of incubation at room temperature (in dark), absorbance was measured at 415 nm. Quercetin was used as a standard.

#### Ascorbate and glutathione concentration and redox status

Ascorbate (ASC) and glutathione (GSH) were determined by HPLC analysis. Hundred milligram frozen leaf tissue was extracted with a MagNALyser, in 1 mL of ice-cold 6% (w/v) meta-phosphoric acid, and antioxidants were separated on a reversed phase HPLC column (100 × 4.6 mm Polaris C_18−_A, 3 mm particle size; 40°C) with an isocratic flow rate of 1 ml min^−1^ of elution buffer (2 mM KCl, pH 2.5 adjusted with o-phosphoric acid). The components were quantified using a custom-made electrochemical detector and the purity and identity of the peaks was confirmed using an in-line DAD (SPD-M10AVP, Shimadzu). Reduced antioxidant concentration was determined after reducing with 0.04 MDTT.

#### Enzyme extraction and enzyme activity assays

Around 100 mg frozen leaf tissue was homogenized in 1 mL of K -Phosphate buffer (0.05 M pH 7.0), containing 2% (w/v) polyvinyl pyrrolidone, EDTA (0.4 mM), PMSF (0.2 mM) and ascorbic acid (1 mM). Peroxidase (POX) activity was measured by monitoring the production of purpurogallin at 430 nm (Kumar and Khan, 1982). Catalase (CAT) activity was calculated out of the decrease in H_2_O_2_ concentration, measured at 240 nm (Aebi, 1984). Measuring the inhibition of NBT reduction at 550 nm was used to assay superoxide dismutase (SOD) activity to (Dhindsa et al., 1981). The activity of ascorbate peroxidase (APX), glutathione reductase (GR), glutathione peroxidase (GPX), monodehydroascorbate reductase (MDAR), and dehydroascorbate reductase (DHAR) were assayed according to Murshed et al. (2008).

Soluble protein was determined according to Lowry method (Lowry et al., 1951).

### Data analysis

To identify differences in antioxidant metabolites and enzyme activities, we first performed a four-way ANOVA [with drought (D), location in the growth zone (L), origin of the lines (O) and their tolerance rating (T) as main factors] to identify which factors affect ROS status in the growth zone. Second, we calculated the average concentration and activity levels in the growth zone for each line for each of the three conditions (well-watered, mild, and severe drought stress). Third, we grouped the lines according to their origin and drought tolerance and compared the absolute metabolite concentrations and enzyme activities in the control condition (**Table 3**). This was followed by a comparison of the response patterns along the developmental zones of those concentrations and activities between the lines by means of clustering, using Multi Experiment Viewer (Saeed et al., 2003). The data was mean-centered, which effectively removed global differences in concentration and activity levels (shown in Table S2) between the lines. A hierarchical clustering was performed to compare the magnitude of differences across the growth zone and the response to the treatments between the lines.

### Statistical analysis

A three-way (for the kinematics parameters, Table 1) and a four-way ANOVA (for the biochemical parameters, Table 2) were conducted using SPSS 16.0. Principal Component Analysis (PCA) of all measured biochemical parameters across the 14 maize lines was performed using XLSTAT (an add-in for Microsoft Excel).

**Table 1:** **Kinematic analysis of the effect of drought stress on cell division and cell expansion during the steady-state growth of the fifth leaf of thirteen maize hybrids and the inbred line B73**.

**Parameters**	**% Change**	**tAF**	**tEU**	**tEG**	**AF**	**EU**	**EG**	**B73**	**D**	**T**	**O**	O^*^T	D^*^T	D^*^O	D^*^O^*^T
*LL* (mm)	C–MD	−17 ± 1	−21 ± 1	−15 ± 3	−20 ± 6	−17 ± 1	−16 ± 2	−17	**0.00**	**0.00**	**0.00**	**0.00**	**0.00**	**0.00**	**0.00**
	C–SD	−47 ± 2	−53 ± 3	−36 ± 2	−53 ± 1	−51 ± 2	−55 ± 6	−40							
*LER* (mm/h)	C–MD	−21 ± 3	−21 ± 0	−17 ± 2	−20 ± 5	−24 ± 3	−25 ± 2	−25	**0.00**	0.06	0.81	**0.00**	0.58	0.66	0.14
	C–SD	−50 ± 1	−42 ± 5	−40 ± 6	−55 ± 1	−62 ± 10	−58 ± 2	−59							
*l*_mat_ (μm)	C–MD	−12 ± 0	−7 ± 4	+1 ± 5	−9 ± 3	−7 ± 13	−8 ± 3	−6	**0.00**	0.75	**0.00**	**0.00**	0.25	0.76	0.36
	C–SD	−20 ± 3	−19 ± 6	−12 ± 4	−26 ± 1	−11 ± 1	−19 ± 3	−13							
*P* (cells/h)	C–MD	−9 ± 11	−5 ± 5	−15 ± 20	−11 ± 1	−34 ± 10	−16 ± 6	−23	**0.00**	**0.00**	0.10	**0.01**	0.96	0.06	**0.04**
	C–SD	−58 ± 15	−48 ± 6	−44 ± 3	−52 ± 12	−59 ± 11	−63 ± 6	−59							
*N*_mer_	C–MD	−5 ± 9	−3 ± 1	−13 ± 4	−9 ± 4	−1 ± 5	−24 ± 3	−21	**0.01**	**0.03**	**0.00**	**0.00**	**0.00**	**0.00**	0.43
	C–SD	−3 ± 11	+4 ± 9	−22 ± 9	−28 ± 3	+13 ± 6	−57 ± 10	−32							
*D* (cell/cell/h)	C–MD	−1 ± 22	−2 ± 7	+10 ± 38	−2 ± 4	−41 ± 14	+5 ± 2	−10	**0.00**	0.64	**0.00**	**0.03**	0.05	0.18	0.73
	C–SD	−57 ± 11	−50 ± 1	−18 ± 10	−33 ± 21	−64 ± 8	−2 ± 37	−45							
*T*_c_ (h)	C–MD	+7 ± 25	+2 ± 6	+8 ± 29	+3 ± 4	+31 ± 4	−6 ± 5	+8	**0.00**	0.30	**0.00**	**0.00**	0.79	**0.00**	**0.03**
	C–SD	+159 ± 69	+100 ± 6	+43 ± 29	+84 ± 70	+195 ± 68	+47 ± 49	+85							
*T*_div_ (h)	C–MD	+25 ± 6	−1 ± 8	+7 ± 28	+19 ± 5	+17 ± 14	−10 ± 5	+6	**0.00**	0.43	**0.00**	**0.00**	0.58	**0.00**	**0.02**
	C–SD	+240 ± 153	+82 ± 23	+39 ± 32	+213 ± 84	+135 ± 64	+31 ± 46	+73							
*L*_mer_ (mm)	C–MD	−8 ± 0	−3 ± 2	−9 ± 8	−16 ± 0	−13 ± 12	−20 ± 5	−23	**0.00**	**0.00**	**0.00**	0.10	**0.00**	0.13	0.61
	C–SD	−18 ± 7	−9 ± 10	−28 ± 13	−39 ± 1	−36 ± 11	−57 ± 7	−23							
*R*_el_ (μm/μm/h)	C–MD	−20 ± 2	−3 ± 4	+8 ± 9	−15 ± 1	−8 ± 6	−4 ± 7	−20	**0.00**	0.35	**0.00**	**0.00**	0.98	**0.00**	0.06
	C–SD	−65 ± 17	−50 ± 9	−40 ± 1	−64 ± 8	−29 ± 8	−61 ± 10	−40							
*T*_el_ (h)	C–MD	+6 ± 27	+1 ± 6	−12 ± 11	+1 ± 4	+34 ± 2	−1 ± 13	+36	**0.00**	0.43	**0.00**	**0.00**	0.58	**0.00**	**0.02**
	C–SD	+157 ± 65	+101 ± 3	+63 ± 2	+74 ± 66	+200 ± 67	+190 ± 93	+198							

*Data are grouped by origin and drought tolerance rating and complemented by the reference inbred line B73 (for individual measurements of the lines see Table S1). Data for the hybrids are mean values for the respective groups +/– SE (n = 2 for tAF, tEU, AF, EU and tEG, and n = 3 for EG, with at least 5 replicate plants per line; data for the individual lines are provided in Table S1) and average only for B73. Data for the European and African hybrids, and B73 were published before (Avramova et al., 2016). A three-way ANOVA on the differential values between control and mild and between control and severe drought conditions, respectively, was used as a statistical test and p-values for the three factors, drought treatment (D), tolerance rating (T), and region of origin (O), as well as the interaction factors, are listed. Significant differences (p < 0.05) are marked in bold. Maize lines: South-African hybrids (AF), Western-European hybrids (EU), Egyptian hybrids (EG), drought-tolerant lines (t); Parameters: Leaf Length (LL), Leaf Elongation Rate (LER), Mature cell length (l_mat_), Cell production rate (P), Cell division rate (D), Cell cycle duration (T_c_), Time in division zone (T_div_), Length of the meristem (L_mer_), Number of cells in the meristem (N_mer_), Cell elongation rate (R_el_), Time in elongation zone (T_el_), Control conditions (C), Mild drought conditions (MD), Severe drought conditions (SD); ANOVA factors: drought treatment (D), drought tolerance (T), origin of the lines (O)*.

**Table 2:** **A four-way ANOVA analysis of oxidative stress determinants, antioxidant molecules and enzyme activities**.

**Parameters**	**D**	**L**	**O**	**T**	D^*^L	D^*^O	D^*^T	L^*^O	L^*^T	O^*^T	D^*^L^*^O	D^*^L^*^T	D^*^O^*^T	L^*^O^*^T	D^*^L^*^O^*^T
MDA	**0.000**	**0.001**	**0.000**	0.551	**0.025**	**0.000**	**0.000**	**0.003**	0.577	0.351	0.165	0.976	**0.000**	0.912	0.707
H_2_O_2_	**0.000**	**0.000**	**0.000**	**0.000**	0.292	**0.000**	**0.000**	0.310	0.685	**0.000**	0.998	0.991	**0.000**	0.671	0.991
FRAP	**0.000**	**0.000**	**0.000**	0.325	**0.018**	**0.000**	0.987	0.352	0.787	**0.003**	0.927	0.999	**0.000**	0.864	1.000
Polyphenols	**0.000**	0.969	**0.000**	**0.000**	1.000	**0.000**	0.104	1.000	1.000	**0.000**	1.000	1.000	0.130	1.000	1.000
Flavonoids	**0.000**	0.910	**0.000**	**0.000**	0.998	**0.007**	0.123	1.000	1.000	**0.000**	1.000	1.000	0.219	1.000	1.000
tASC	**0.000**	**0.000**	**0.000**	**0.000**	**0.002**	**0.000**	**0.000**	**0.001**	0.268	**0.000**	**0.011**	0.922	**0.000**	0.063	1.000
ASC	**0.018**	**0.000**	**0.000**	**0.000**	0.181	**0.000**	0.083	**0.037**	**0.002**	**0.000**	0.959	0.762	**0.000**	0.890	0.945
tGSH	**0.000**	**0.000**	**0.000**	**0.000**	**0.018**	**0.000**	**0.000**	0.695	0.956	**0.000**	0.668	0.998	**0.000**	0.752	1.000
GSH	**0.000**	**0.000**	**0.000**	0.106	0.342	**0.000**	**0.011**	**0.006**	0.898	**0.000**	0.989	0.991	**0.005**	0.111	1.000
SOD	**0.033**	0.132	**0.000**	**0.000**	0.911	**0.010**	**0.000**	0.261	0.838	**0.000**	0.311	0.780	**0.000**	0.651	0.914
CAT	**0.001**	**0.000**	**0.000**	**0.000**	**0.002**	**0.000**	0.081	**0.000**	**0.000**	**0.000**	**0.000**	0.139	**0.000**	**0.000**	**0.033**
POX	**0.000**	**0.000**	**0.000**	**0.000**	0.292	**0.000**	**0.002**	**0.000**	**0.007**	0.451	0.746	0.822	0.569	**0.019**	0.867
APX	**0.000**	**0.000**	**0.000**	**0.000**	**0.043**	**0.005**	**0.000**	**0.000**	**0.022**	**0.000**	0.321	0.569	**0.000**	**0.000**	0.744
DHAR	**0.000**	**0.000**	**0.000**	**0.000**	**0.000**	**0.000**	**0.000**	0.286	0.221	**0.000**	0.652	0.613	**0.000**	0.108	0.072
MDAR	**0.000**	**0.010**	**0.000**	**0.000**	**0.017**	0.543	0.056	**0.000**	0.056	**0.000**	**0.000**	0.052	**0.000**	**0.043**	**0.021**
GR	**0.000**	**0.000**	**0.000**	**0.000**	0.797	**0.000**	**0.000**	**0.000**	**0.000**	**0.000**	**0.000**	**0.009**	**0.015**	**0.000**	**0.004**
GPX	0.409	0.873	**0.000**	0.944	0.999	0.100	0.065	0.950	0.945	**0.000**	1.000	1.000	0.218	0.578	1.000

*The table lists the significance of the effects of region of origin (O) of the 13 maize hybrids, drought treatment (D), location in the growth zone of the leaf (L), and differences in drought tolerance (T) between the hybrids (breeder's ranking, Avramova et al., 2016) and the interaction between them. Significant differences (p < 0.05) are marked in bold. MDA, malondialdehyde; ASC, ascorbate; tASC, total ascorbate; GSH, glutathione; tGSH, total glutathione; FRAP, ferric reducing ability of plasma; SOD, superoxid dismutase; CAT, catalase; POX, peroxidase; APX, ascorbate peroxidase; DHAR, dehydroascorbate reductase; MDAR, monodehydroascorbate reductase; GPX, glutathion peroxidase; GR, gluthation reductase*.

## Results

### Leaf growth

We studied the growth of 13 hybrid maize lines with different geographical backgrounds (Western Europe, Egypt, and South Africa) and contrasting drought tolerance, and the reference inbred line B73 under optimal, mild (no leaf wilting), and severe (leaf wilting) drought conditions. In B73 the mild and severe treatments inhibited leaf elongation rate by 27 and 63%, respectively, but did not cause senescence (Avramova et al., 2015a). Half of the European and the African hybrids were rated as drought tolerant (see Avramova et al., 2016). In spite of the fact that the hybrid maize lines originated from different continents, they were all provided by the same breeding company (Pioneer) and consequently from related breeding programs. Therefore, the overall variation between these hybrids was relatively small (Avramova et al., 2016). In this study, we add the analysis of 5 Egyptian lines, obtained from an independent breeding program (Sids Research Station, Agricultural Research Centre, Beni-Suef, Egypt) to the kinematic analysis of the African and the European hybrids, and B73, published in our previous study (Avramova et al., 2016) to broaden the genotypic variation and compare the behavior of these groups of lines with different drought tolerance and different origins.

The response to drought and salinity stress is often similar with respect to physiological, biochemical, molecular and genetic effects (Sairam and Tyagi, 2004). Two of the Egyptian lines (EG) were rated as tolerant to salt stress and the other three were more sensitive (unpublished data, Sids Research Station, Agricultural Research Centre, Beni-Suef, Egypt). Therefore, we expected salinity-tolerant lines to be more drought-tolerant than the rest of the lines. Indeed, under our water-deficient conditions the salt-tolerant lines showed a smaller reduction of leaf elongation rate in both mild and severe treatments (Table S1) than the other three Egyptian lines. Therefore, in this study, we designated them also as drought-tolerant.

On average, the final leaf length (*LL*) was reduced by 17% in response to mild stress and 50% in response to the severe stress conditions. However, *LL* of the tolerant lines was significantly less affected by the severe stress (−45 and −53% respectively, Table 1; Table S1). Lines from different origins showed significant differences for *LL*, the leaves of the B73 (727 mm) and the Egyptian lines (916 ± 46 mm) being significantly shorter than the European (1008 ± 23 mm) and South African lines (983 ± 14 mm; Table S1). Most interestingly, a three-way ANOVA (factors: Drought treatment, D; drought tolerance, T; and origin of the maize line, O) showed a highly significant O*T factor, related to the tolerant Egyptian lines (tEG) being significantly less affected by the drought (−15 and −36%, in response to mild and severe drought) than the tolerant lines from the other origins (−17 to −21 and −47 to −53%, in response to mild and severe drought; Table 1, Table S1). B73 behaved differently from the hybrids, showing one of the largest reductions in leaf size during the mild stress (−17%), but one of the smallest reductions in the severe stress conditions (−40%). Leaf elongation rates (*LER*) of all the lines were reduced more than *LL*, demonstrating that compensation by increased duration of the leaf growth occurs. Nevertheless, differences in *LER* correlate and explain most of the variation in *LL* (Table 1).

To understand the cellular basis of the leaf growth response we performed a kinematic analysis. *LER* is a function of cell production in the meristem and mature cell length determined in the elongation zone. Therefore, the effect of drought must be due to effects on one or both of these parameters. Our results show that both cell production rate (*P*) and mature cell length (*l*_*mat*_) were reduced by mild and severe drought conditions. Cell production was more sensitive, particularly to the severe treatment. The three-way ANOVA analysis showed significant differences in cell production rate, but not in cell length, linked to tolerance rating (Table 1). Cell production in the tolerant hybrids from all the regions was less affected than in the other lines, tEG being the least affected by the severe drought (−44%), while the contrasting Egyptian hybrids (EG) showing the strongest reduction (−63%).

Cell production, in turn, depends on the cell division rate (*D*; inversely proportional to cell cycle duration, *T*_*c*_) and number of cells in the division zone, (*N*_*mer*_, which closely relates to meristem length, *L*_*mer*_). Across all lines and in both treatments, drought had a roughly equal effect on both parameters. Strikingly, the reductions in meristem length (−7 vs. −29% for tolerant and non-tolerant lines under severe drought) and number of cells in the meristem (−18 vs. −46% for tolerant and non-tolerant lines under severe drought) were significantly smaller in the tolerant lines from all origins, which contributed to the smaller decrease in cell production in these lines. On the other hand, hybrids from different origins also exhibited different mechanisms to respond to the stress. In the Egyptian lines (both tEG and EG) cell division rates (−18 and −2% for tEG and EG under severe drought and its inverse, cell cycle duration, +43 and 47% for tEG and EG under severe drought) and time in the division zone were least affected. In contrast, these lines showed the largest reduction in meristem length (−28 and −57 for tEG and EG under severe drought) and number of cells in the meristem (−22 and −57% for tEG and EG under severe drought). The EG lines were significantly more sensitive than the tEG, according to these parameters. Inversely, in the EU lines cell division rate (and cell cycle duration) was more strongly affected by the drought (−21 and −57% under mild and severe drought, respectively). This was partly compensated by a smaller reduction in meristem length (−8 and −22% under mild and severe drought, respectively) and no significant effect on the number of cells in the meristem, leading to a smaller decrease in cell production rate (−19 and −52% under mild and severe drought), compared to the non-tolerant EG lines (−16 and −63% under mild and severe drought, respectively).

Although the mature cell length (*l*_*mat*_) contributed less than the cell production rate to the reduction in *LER*, the average cell expansion rate (*R*_*el*_) was significantly (between −29 and −65%) reduced by severe drought in all lines (Table 1). However, this reduction was compensated by a significant increase in the time the cells spent in the elongation zone (*T*_*el*_, between +63 and +200%). There were no significant differences between the tolerant and the other hybrids according to these parameters. However, *R*_*el*_(−65%) and consequently *l*_*mat*_(−20% and −26%, for tAF and AF, respectively) were affected the most in the South African hybrids. Inversely, both parameters were the least decreased by the drought in the tEG hybrids (*R*_*el*_ −40%; *l*_*mat*_ −12%) in the severe stress conditions and even slightly increased (*R*_*el*_ +8%; *l*_*mat*_ +1%) in the mild stress conditions. This, in combination with their smallest reduction in cell division rate (*D;* −18% in response to severe drought), explains why overall these hybrids exhibit the highest degree of drought tolerance.

We conclude that, as expected, leaf growth of tolerant maize hybrids was in general less affected by the drought, which was largely due to smaller reductions in cell production rate, meristem size and number of cells in the meristem. The tEG lines were least affected by the drought stress conditions in terms of cell division and cell elongation rates, while the non-tolerant EG lines were the most sensitive, particularly to the severe stress (Table 1).

### Redox status and antioxidant content in the maize leaf

Next, we set out to investigate if differences in stress defenses in the leaf growth zone could be related to the differences in cellular responses to drought. To this end we measured H_2_O_2_ and MDA levels, to estimate oxidative stress and membrane damage, respectively, as well as total antioxidant capacity, antioxidant metabolites (polyphenols, flavonoids, ascorbate, and glutathione), and antioxidant enzyme activities (catalase, CAT; peroxidases, POX; superoxide dismutase, SOD; ascorbate peroxidase, APX; dehydroascorbate reductase, DHAR; monodehydroascorbate reductase, MDAR; glutathione reductase, GR; and glutathione peroxidase, GPX). As the maize-leaf model system allows biochemical analysis across the developmental zones, we measured all parameters in 1 cm segments, spanning the meristem (first 1–2 cm from the leaf base), the elongation zone (next 4–5 cm), and mature tissue (remainder of the leaf; Table S1). Note that the kinematic analysis shows differences in the size of meristem and elongation zone between treatments and lines, so that the same spatial position does not necessarily correspond to the same developmental stage. Nevertheless, sampling at 10 positions along the growth zone allows comparing the same developmental stage in terms of metabolite concentrations and enzyme activities in all line/condition combinations.

**Figure 1:**
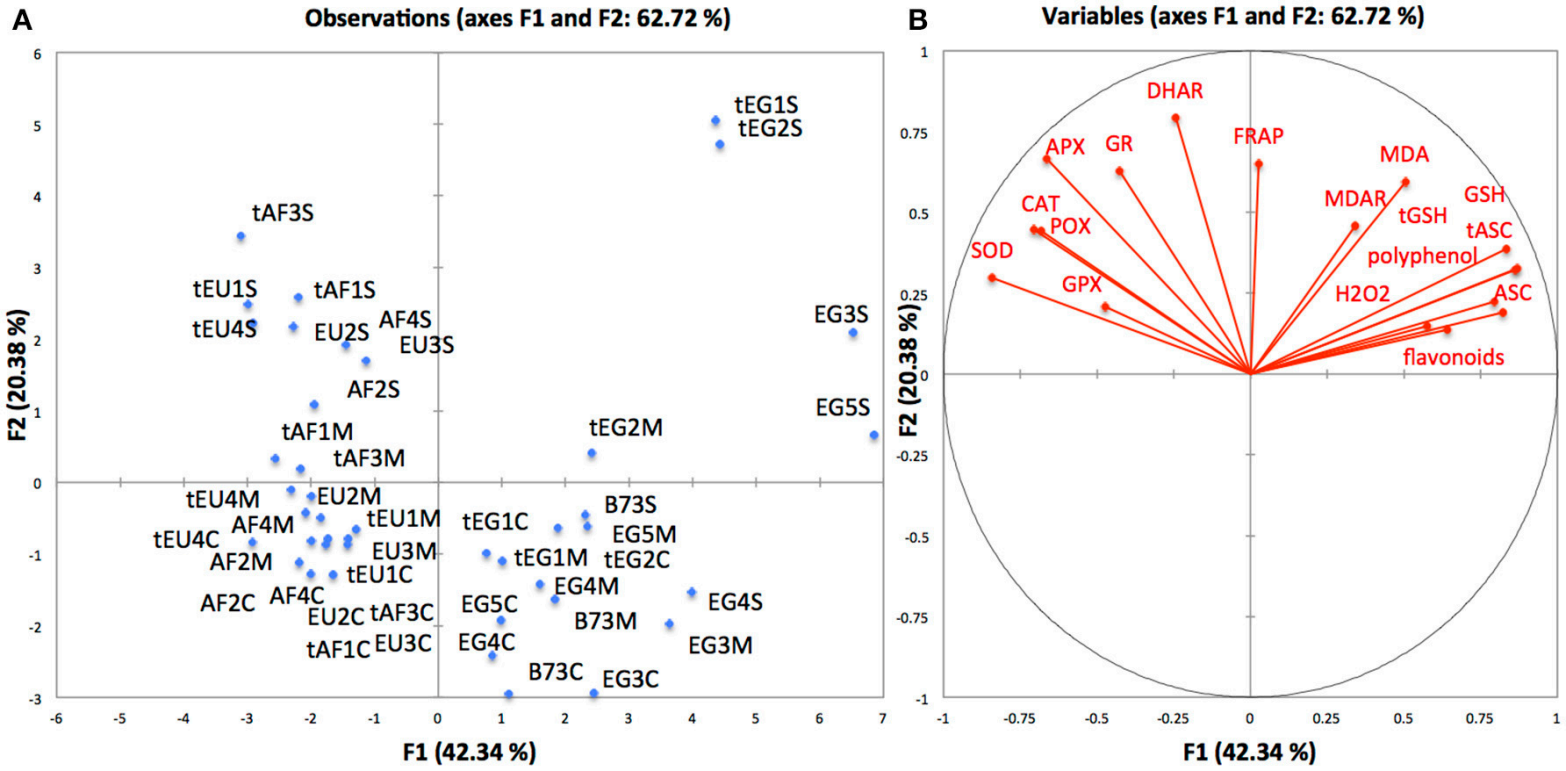
**Principal component analysis of variation in antioxidant metabolite levels and enzyme activities across maize lines in response to drought**. Data for 14 lines grown under control (marked with C), mild (marked with M), and severe (marked with S) drought. The lines are grouped according to antioxidant concentrations and enzyme activities **(A)** and the explanatory variables **(B)**. EU-European hybrids, AF-African hybrids, EG- Egyptian hybrids, t-drought tolerant line.

To get a global view of the data, we first performed a Principal Component Analysis (PCA; Figure 1) allowing to observe global patterns of the responses of the contrasting maize lines according to oxidative stress and antioxidant parameters, and to evaluate to what extent this grouping corresponds to the observed variations in growth cellular parameters. PC1, accounting for 42% of the data variation, separated the maize lines in two main groups: African with European hybrids, and Egyptian hybrids with B73. This was consistent with the kinematics results, showing that the Egyptian lines and B73 differed most from all other hybrids. Interestingly, the enzyme activities and the metabolite concentrations separated the lines in contrasting ways. Metabolites are determinant for the position of the Egyptian and B73 group, whereas the enzymes determined the direction of the European and African hybrids (Figure 1). This suggests different molecular mechanisms in each group. PC2, which accounts for 20% of the variation, separates the treatments (control, C; mild stress, M; severe stress, S). The control conditions and the severe stress were clearly separated, while the mild drought grouped together with the severe stress for the lines tAF1, tAF3, and tEG2, and together with the controls for the rest of the lines, consistent with its intermediate character. There was no clear pattern in grouping of the African and the European hybrids, based on their drought sensitivity rating. The spread among the Egyptian lines is clearly larger than among the European and African lines, the treatment effect (PC2) being the most pronounced in tEG1 and tEG2 under severe stress (Figure 1). As the growth of these two lines was the least affected (Table 1), their tolerance could therefor potentially be explained by their antioxidant concentrations and enzyme activities.

**Figure 2:**
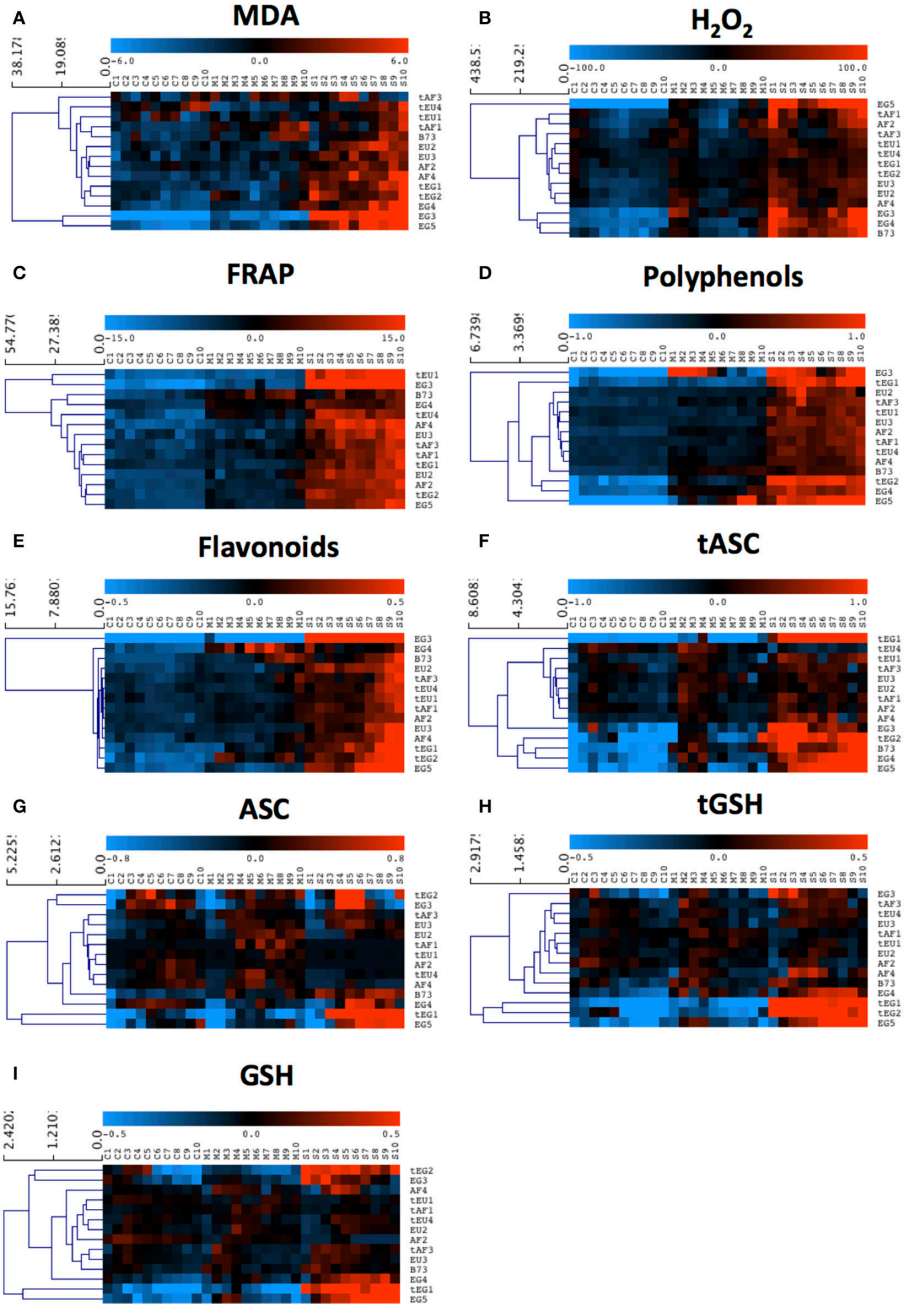
**The response of antioxidant metabolite concentrations in the growth zone to drought**. Metabolite concentrations were measured in each centimeter (from 1 to 10) of the growth zone of the 5th leaf of plants grown in well-watered conditions (C1–C10) and subjected to mild (M1–M10) and severe (S1–S10) drought stress. Three biological replicates (each consisting of 2–3 pooled plants) were measured for each line and the data are presented as averages. Data were mean-centered to remove differences in absolute levels (shown in Table 3 and Table S2) and hierarchically clustered to show patterns across the growth zone and responses to the drought. **(A)** Malondialdehyde (MDA), **(B)** Hydrogen peroxide (H_2_O_2_), **(C)** Ferric Reducing Ability of Plasma (FRAP), **(D)** Polyphenols, **(E)** Flavonoids, **(F)** total ascorbate (tASC), **(G)** total glutathione (tGSH), **(H)** reduced ascorbate (ASC), **(I)** reduced glutathione (GSH).

**Figure 3:**
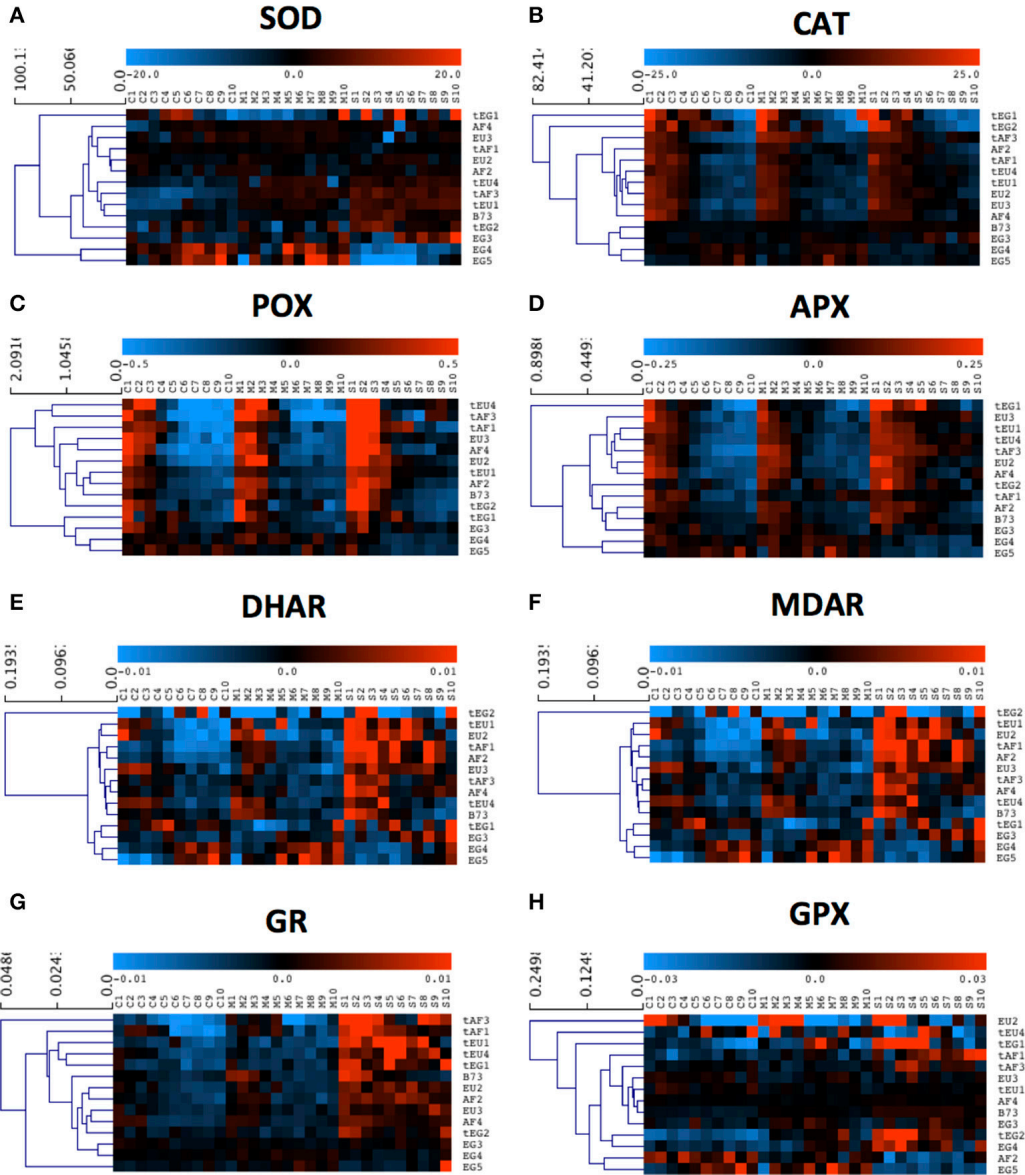
**The response of activities of the main redox-regulating enzymes to drought**. Enzyme activities were measured in each centimeter (from 1 to 10) of the growth zone of the 5th leaf of plants, grown in well-watered conditions (C1–C10) and plants, subjected to mild (M1–M10) and severe (S1–S10) drought stress. Three biological replicates (each consisting of 2–3 pooled plants) were measured for each line and the data are presented as averages. Data were mean-centered to remove differences in absolute levels (shown in Table 3 and Table S2) and hierarchically clustered to show patterns across the growth zone and responses to the drought. **(A)** Superoxide dismutase (SOD), **(B)** Catalase (CAT), **(C)** Peroxidase (POX), **(D)** Ascorbate reductase (APX), **(E)** Dehydroascorbate reductase (DHAR), **(F)** Monodehydroascorbate reductase (MDAR), **(G)** Glutathione reductase (GR), **(H)** Glutathione-S-transferase (GPX).

To explore the basis of the different response of the maize lines and the relation between cellular growth parameters and molecular antioxidant mechanisms, we studied their response to drought across the growth zone in terms of stress-induced oxidative damage and ROS status. We performed a four-way ANOVA to test for statistically significant contrasts, with drought (D), location in the growth zone (L), geographic origin (O), and tolerance rating (T) as factors (Table 2). Correlations between tolerance and various antioxidant levels, were tested by grouping the maize lines according to their origin and drought tolerance, and comparing to the metabolite concentrations and enzyme activities in the control condition (Table 3). Responsiveness to drought across the developmental zones was evaluated by comparing these levels in control, mild and sever conditions using a hierarchical clustering of mean-centered data, which effectively removed global differences in concentration and activity levels (shown in Table S2) between the lines (Figures 2, 3).

**Table 3:** **The concentration of oxidative stress determinants, antioxidant molecules, and enzyme activities**.

**Maize lines**	**MDA (μmol/gFW)**	**H_2_O_2_(nmol /mg FW)**	**FRAP (μlTrolox/gFW)**	**Polyphenols (mg GA/g FW)**	**Flavonoids (mg QA/g FW)**	**tASC(μmol/gFW)**	**ASC (μmol/gFW)**	**tGSH(μmol/gFW)**	**GSH (μmol/gFW)**
tEU	7 ± 0	68 ± 2	12 ± 1	0.39 ± 0.01	0.21 ± 0.01	1.1 ± 0.0	0.1 ± 0.0	0.3 ± 0.0	0.2 ± 0.04
tAF	5 ± 0	95 ± 5	13 ± 0	0.43 ± 0.01	0.24 ± 0.01	0.8 ± 0.0	0.2 ± 0.0	0.2 ± 0.0	0.1 ± 0.0
EU	6 ± 0	58 ± 3	13 ± 1	0.38 ± 0.01	0.22 ± 0.01	1 ± 0.0	0.3 ± 0.0	0.4 ± 0.0	0.3 ± 0.0
AF	5 ± 0	82 ± 4	11 ± 0	0.39 ± 0.01	0.20 ± 0.02	1 ± 0.0	0.2 ± 0.0	0.4 ± 0.0	0.3 ± 0.0
B73	3 ± 0	150 ± 9	15 ± 1	0.40 ± 0.01	0.25 ± 0.02	2 ± 0.3	0.6 ± 0.1	0.4 ± 0.0	0.3 ± 0.0
tEG	7 ± 0	103 ± 3	7 ± 0	0.84 ± 0.04	0.44 ± 0.02	2.2 ± 0.1	1.3 ± 0.1	0.9 ± 0.1	0.8 ± 0.1
EG	4 ± 1	87 ± 4	8 ± 0	1.53 ± 0.17	1.04 ± 0.16	1.8 ± 0.1	1.0 ± 0.1	0.6 ± 0.0	0.6 ± 0.0
Tolerant hybrids	6 ± 1	89 ± 7	10 ± 1	0.55 ± 0.09	0.30 ± 0.05	1.3 ± 0.27	0.5 ± 0.25	0.5 ± 0.14	0.4 ± 0.14
Non-tolerant hybrids	5 ± 1	77 ± 7	10 ± 1	0.88 ± 0.32	0.56 ± 0.28	1.3 ± 0.18	0.6 ± 0.16	0.5 ± 0.06	0.4 ± 0.07
EU hybrids	6 ± 1	63 ± 3	12 ± 2	0.39 ± 0.02	0.21 ± 0.02	1.0 ± 0.03	0.2 ± 0.05	0.3 ± 0.04	0.2 ± 0.04
AF hybrids	5 ± 1	88 ± 7	12 ± 1	0.41 ± 0.02	0.22 ± 0.02	0.9 ± 0.06	0.2 ± 0.06	0.3 ± 0.06	0.2 ± 0.05
EG hybrids	5 ± 1	93 ± 9	7 ± 1	1.25 ± 0.37	0.80 ± 0.36	1.9 ± 0.16	1.1 ± 0.09	0.7 ± 0.08	0.7 ± 0.07
**Maize lines**	**SOD (Unit /mg protein.min)**	**CAT (**μ**mol H**_2_**O**_2_**/mg protein.min)**	**POX (**μ**mol oxidized pyrogallol/mg protein.min)**	**APX (**μ**mol ASC/mg protein.min)**	**DHAR (nmol ASC/mg protein.min)**	**MDAR (**μ**mol NADH/mg protein.min)**	**GR (**μ**mol NADPH/mg protein.min)**	**GPX (**μ**mol NADPH/mg protein.min)**	
tEU	50 ± 1	25 ± 2	0.7 ± 0.1	0.27 ± 0.02	7.2 ± 0.3	0.012 ± 0.001	0.007 ± 0.000	0.093 ± 0.016	
tAF	59 ± 3	26 ± 2	0.8 ± 0.1	0.26 ± 0.02	5.8 ± 0.2	0.006 ± 0.000	0.011 ± 0.001	0.067 ± 0.002	
EU	59 ± 2	25 ± 2	0.6 ± 0.1	0.27 ± 0.02	4.9 ± 0.2	0.015 ± 0.001	0.004 ± 0.000	0.110 ± 0.013	
AF	62 ± 2	25 ± 2	0.7 ± 0.1	0.26 ± 0.02	5.1 ± 0.3	0.005 ± 0.000	0.006 ± 0.000	0.040 ± 0.008	
B73	29 ± 1	3 ± 0	0.4 ± 0.0	0.14 ± 0.01	3.8 ± 0.2	0.011 ± 0.000	0.007 ± 0.000	0.013 ± 0.000	
tEG	38 ± 3	28 ± 3	0.4 ± 0.0	0.20 ± 0.02	4.5 ± 0.2	0.026 ± 0.002	0.005 ± 0.000	0.015 ± 0.002	
EG	29 ± 2	11 ± 1	0.4 ± 0.0	0.20 ± 0.01	3.5 ± 0.3	0.014 ± 0.000	0.003 ± 0.000	0.032 ± 0.002	
Tolerant hybrids	49 ± 6	26 ± 1	0.6 ± 0.0	0.24 ± 0.02	5.8 ± 0.6	0.015 ± 0.004	0.007 ± 0.001	0.058 ± 0.022	
Non-tolerant hybrids	47 ± 7	19 ± 3	0.6 ± 0.1	0.24 ± 0.02	4.4 ± 0.4	0.012 ± 0.002	0.004 ± 0.001	0.056 ± 0.020	
EU hybrids	54 ± 4	25 ± 1	0.7 ± 0.1	0.27 ± 0.01	6.0 ± 0.8	0.014 ± 0.001	0.005 ± 0.001	0.101 ± 0.034	
AF hybrids	61 ± 5	25 ± 2	0.8 ± 0.0	0.26 ± 0.01	5.4 ± 0.2	0.005 ± 0.000	0.008 ± 0.002	0.053 ± 0.017	
EG hybrids	32 ± 6	18 ± 4	0.4 ± 0.0	0.20 ± 0.01	3.9 ± 0.4	0.019 ± 0.004	0.004 ± 0.001	0.025 ± 0.005	

*Data are averaged along the growth zones of leaves from well-watered seedlings of hybrids with different drought sensitivity and origin, and the inbred line B73. Leaf growth zones were divided in 10 equal segments and measurements were performed in each of the 10 segments. Three biological replicates (each consisting of 2–3 pooled plants) were measured for each line. Data are averages across all 10 segments of the growth zones and across the lines in the indicated group +/– SE (n = 20; for B73 n = 10; for EG n = 30). MDA, malondialdehyde; ASC, ascorbate; tASC, total ascorbate; GSH, glutathione; tGSH, total glutathione; FRAP, ferric reducing ability of plasma; SOD, superoxid dismutase; CAT, catalase; POX, peroxidase; APX, ascorbate peroxidase; DHAR, dehydroascorbate reductase; MDAR, monodehydroascorbate reductase; GPX, glutathion peroxidase; GR, gluthation reductase; FW, fresh weight; GA, gallic acid; QA, quercetin*.

We first analyzed malondialdehyde (MDA), a secondary metabolite resulting from lipid peroxidation by ROS, reflecting oxidative damage levels (Picaud et al., 2004). Differences in MDA levels were significant for 8 out of total 15 factors and between-factors interactions (Table 2), demonstrating the impact of drought (significant D), and also demonstrating that the different regions of the growth zone (significant L), and maize lines from different geographic origin (significant O), experienced varying extents of lipid peroxidation. Moreover, lines from different origins and tolerance rating responded differently to the drought (significant D*O, D*T, D*O*T interactions; Table 2). In control conditions, the tEU and tEG hybrids had the highest MDA levels in their growth zones, and the EG hybrids and B73 the lowest (Table 3). However, the line with the highest MDA absolute levels under control conditions (tEU4) did not increase those levels during the stress (8.1 μmol/gFW, Figure 2A, Table S2). In contrast, the line EG3 had the lowest MDA concentration under control conditions (1.8 μmol/gFW) and the highest MDA concentration under severe stress conditions (19 μmol/gFW; Figure 2A, Table S2). MDA levels increased particularly in the mature part of the leaf, with the severity of the stress, but to a different extent for the different lines (Figure 2A). Overall, while in tolerant lines, tEU1, tEU4, tAF1, and tAF3 MDA levels were only slightly affected by drought, in non-tolerant lines and B73, they roughly doubled (reaching the highest values for the lines EG3, EG4, EG5) in the severe stress conditions (Figure 2A). Therefore, we conclude that the MDA contents across the growth zone of drought tolerant lines were somewhat higher in control conditions, but changed less in response to drought. Inversely, lines that performed less well under drought had low MDA levels under control conditions, but showed a more pronounced increase in response to the stress.

Most reactive oxygen species are short-lived, extremely reactive, and therefore hard to quantify. However, methods for reliable quantitative H_2_O_2_ detection have been established (Bellincampi et al., 2000), and a comparison of such methods demonstrated their usefulness to reliably measure H_2_O_2_ in the maize leaf growth zone (Avramova et al., 2015a). H_2_O_2_ levels were significantly different for 8 ANOVA factors and between-factor interactions (Table 2), largely overlapping with the changes in MDA concentrations, reflecting that ROS levels correlate with oxidative damage. Conspicuously, there were no interaction effects with location in the growth zone (interactions with L) indicating that, although H_2_O_2_ levels vary along the growth zone (significant L, Figure 2B), they are affected proportionally by drought and genotype across the growth zone. In control conditions, the lowest H_2_O_2_concentrations were measured in the EU lines (58 ± 3 nmol/mg FW) and the highest in B73 (150 ± 9 nmol/mg FW; Table 3), followed by the tEG hybrids (103 ± 3 nmol/mg FW). Overall, the tolerant lines had higher H_2_O_2_ levels in the control conditions (significant T in the ANOVA analysis; Table 2) than the rest of the lines (Table 3, Table S2). Generally, H_2_O_2_ concentrations progressively increased across the growth zone with increasing stress levels. However, the tolerant lines showed relatively smaller changes in the stress conditions compared to the rest of the lines (Figure 2B). The elongation zone consistently had the lowest H_2_O_2_ content. The accumulation patterns were markedly different between the European and the African hybrids on one hand, and the Egyptian lines, on the other hand. The two tEG lines accumulated relatively high amounts of H_2_O_2_ in the meristem in the well-watered conditions (Table 3), while under drought conditions H_2_O_2_ levels increased throughout the leaf (Figure 2). In contrast, in the 3 EG lines, there was less H_2_O_2_ accumulation in the leaves under well-watered conditions, but a stronger accumulation occurred in the drought conditions. The hybrids, labeled as tolerant, clustered close to each other and the inbred line B73 clustered together with two of the most sensitive Egyptian hybrid lines (EG3 and 4; Figure 2B). Therefore, similar to the MDA results, drought tolerance correlates with higher H_2_O_2_ levels in control conditions and a relatively small increase in response to the stress.

Besides its oxidative effect, H_2_O_2_ (and other ROS) play a role as a signaling molecule (Veal and Day, 2011). Its accumulation during stress initiates the response of the plant defense system, including the accumulation of antioxidants and activation of redox-regulating enzymes. To understand the basis of protection against rising ROS levels in the leaf growth zone, we measured overall antioxidant capacity and the content of important antioxidant molecules using the Ferric Reducing Ability of Plasma (FRAP) assay (Benzie and Strain, 1996). The ANOVA analysis showed significant differences for 7 factors and between-factor interactions, demonstrating that the origin of the lines (significant O) is one of the main factors determining global differences in total antioxidant capacity as well as the response of these levels to drought (significant D; Table 2). This was mainly due the Egyptian hybrids having the lowest total antioxidant capacity in the control conditions (7–8 μlTrolox/gFW Table 3), but the highest increase during the stress conditions (increasing to 25 μlTrolox/gFW under severe drought; Figure 2C, Table S2). B73 had the highest total antioxidant capacity in control conditions (15 μlTrolox/gFW; Table 3), but those levels did not increase in the severe stress to the same extent as in the hybrid lines (to 24 μlTrolox/gFW; Figure 2, Table S2). Antioxidant levels were approximately stable across the growth zone and increased proportionally with the drought levels (Figure 2C). B73, tEU4, and EG4 differed from the hybrid lines, showing increased capacity under mild drought, which did not increase further under severe drought; Figure 2C). On the other hand, tEU1 and EG3 clustered separately from the rest, having a more pronounced response to the stress than the other lines.

Similar patterns were observed for the polyphenol content, which was significantly different for 5 ANOVA factors and between-factor interactions (Table 2). In terms of drought tolerance, tAF and tEU had higher polyphenol levels (0.43 and 0.39 mg GA/g FW, respectively) in control conditions than AF and EU (0.39 and 0.38 mg GA/g FW, respectively), but the opposite was observed for the tEG and EG (0.83 and 1.53 mg GA/g FW, respectively; Table 3). Polyphenol levels were roughly stable along the leaf axis and progressively increased with the increase in stress severity (Figure 2D). The Egyptian hybrids had higher polyphenol levels than the rest of the lines in the control conditions (1.25 mg GA/g FW; Table 3) and showed the largest increase in response to the stress (to 3.3 mg GA/g FW in severe drought), clustering separately from the rest of the lines (Figure 2D). On the other hand, B73 was the line with the smallest change in polyphenol concentration in response to the stress (increasing from 0.4 in control to 0.9 mg GA/g FW in severe drought conditions; Figure 2D, Table S2). Polyphenol levels in EG3 increased in the meristem under mild stress conditions more strongly than the other lines, while EG5 and to a lesser extent EG4 and B73 showed an increase in the mature zone under mild stress (Figure 2D). In conclusion, the polyphenol measurements show that the Egyptian lines were more responsive to the drought stress.

The flavonoid concentration showed the same tendencies as the polyphenols regarding statistical differences identified by the ANOVA analysis (Table 2) with the Egyptian lines having higher concentrations in control conditions (0.80 mg QA/g FW; Table 3) and a stronger response to the stress (to 2.5 mg QA/g FW; Table S2, Figure 2E). However, the pattern along the growth zone was different (Figure 2E). Clearly, this class of antioxidants increased their concentration toward the mature zone during the severe drought stress, and less so in drought tolerant hybrids, which clustered together. EG3 showed the biggest change in flavonoid concentrations in response to the stress, but the Egyptian lines (especially the non-tolerant EG lines) in general were most responsive to the stress, whereas the tEU and tAF were the least responsive. Tolerant hybrids grouped closely together (Figure 2E) having a weaker response than the non-tolerant lines, showing that the increase in flavonoid levels in response to drought is inversely proportional to drought tolerance.

The ANOVA analysis of ascorbate (ASC) and glutathione (GSH) levels showed significance in 6 factors and between-factor interactions for both total and the reduced concentrations of ASC and GSH, including the main effects drought (D), location in the growth zone (L), and region of origin (O; Table 2). The factor tolerance (T) was significant in terms of tASC, ASC, tGSH, but not for GSH. The concentrations of both total ASC and total GSH were higher in the Egyptian hybrids than in B73, the EU and AF lines in the control conditions (Table 3) and with B73, were more responsive in those lines compared to the rest of the hybrids (Figures 2F,H). The reduced ascorbate (ASC) and the reduced glutathione (GSH) followed the same tendencies (Figures 2G,I, Table S2). There were two main patterns of ASC concentrations along the growth zones (Figure 2G). In most of the African and the European hybrids, the ASC content was higher in the control and the mild drought stress conditions and lower in the severe drought conditions. In the Egyptian hybrids and B73 the opposite pattern occurred: ASC concentrations were the highest in the severe drought conditions. In all the lines, ASC concentrations were lowest in the meristem part of the leaf. Comparing the ASC profiles (Figure 2G) to those of tASC (Figure 2F), it was clear that, across all genotypes, ascorbate was oxidized in the elongation zone. GSH and tGSH (Figures 2H,I) showed similar patterns, with the Egyptian lines clearly separated from the rest of the lines, having higher GSH levels in well-watered conditions (Table 3) and being more responsive to drought conditions (Figure 2H). The patterns of tGSH along the growth zone were similar to those of GSH, suggesting that GSH oxidation was not specifically localized at a certain developmental stage along the leaf axis.

### Enzyme activities

Various ROS scavenging enzymes, contribute to the redox status control of plants during environmental stress. Superoxide dismutase (SOD) was significantly affected for 7 factors and between-factor interactions in the ANOVA, including the main effects drought (D), region of origin (O), and tolerance rating (T). The absence of a significant effect of location (L; Table 2), indicates that the levels are stable across the growth zone (Figure 3A). Differences between lines of different origin were clear in the control conditions, as SOD was less active in the leaves of B73 and the EG hybrids (both 29 units/mg protein.min; Table 3). Overall, the tolerant hybrids had similar SOD activity in the control conditions (49 vs 47 units /mg protein.min for tolerant and non-tolerant lines, respectively; Table 3). For the majority of the lines, the activity of the enzyme increased proportionally to the severity of the stress, except for the non-tolerant Egyptian hybrids (EG4 and EG5), where it decreased in the severe stress conditions compared to the controls (Figure 3A, Table S2). Although SOD activity did not show much variation along the growth zone in control conditions, in most of the tolerant lines, severe drought stress induced the SOD activity specifically in the meristem (Figure 3A).

The activity of H_2_O_2_ scavenging enzymes catalase (CAT), peroxidase (POX), and ascorbate peroxidase (APX), was significantly different for similar ANOVA factors and between-factors interactions, including all main effects: Drought (D), location (L), region of origin (O), and tolerance rating (T), suggesting similarities in the regulation of their activity in the leaf growth zone (Table 2). Indeed, CAT, POX, and APX activities were all higher in the meristem in all conditions, and increased with the stress for the majority of the lines (Figures 3B–D). In control conditions, the activity of the three enzymes was lower in B73 (particularly CAT) and the EG hybrids compared to the rest of the lines and for CAT it was higher in the tolerant hybrids than in the rest of the hybrids (26 vs. 19 μmol H_2_O_2_/mg protein.min; Table 3). The non-tolerant Egyptian lines EG3, EG4, and EG5 behaved differently, showing a virtual absence of a zone effect and response to stress (Figures 3B–D, Table S2).

The differences in the activity of the ASC/GSH regenerating enzymes, dehydroascorbate reductase (DHAR), monodehydroascorbate reductase (MDAR), and glutathione reductase (GR), were significant for nearly all ANOVA factors and between-factor interactions (Table 2). The DHAR, MDAR and GR activities in control conditions were positively correlated with tolerance (5.8 compared to 4.4 nmol ASC/mg protein.min for tolerant and non-tolerant lines, respectively; Table 3). The expression patterns generally showed highest activity at the base of the leaf and an induction proportional to the stress level (Figures 3E–G). In contrast, the EG hybrids had the highest DHAR and MDHAR activity in the mature part of the leaf, a constant level of GR activity across the growth zone and showed no induction of any of these enzymes by the stress (Figures 3E–G). The hierarchical clustering of GR activity showed that drought-tolerant lines more strongly upregulated this activity in response to severe stress (Figures 3E–G).

In contrast to the strong effects on DHAR, MDHAR and GR, differences in GPX activity levels were only significant for the factor geographic origin (O) and the interaction Origin*Tolerance (O*T). This was due to EU hybrids having a nearly 4-fold higher activity than the EG lines (0.101 vs. 0.025 μmol NADPH/mg protein.min; Table 3). Interestingly, the two tEG hybrids had the lowest enzyme activity in the control conditions (0.015 μmol NADPH/mg protein.min; Table 3), but the strongest increase in the severe stress conditions (Figure 3H, Table S2). There was no consistent pattern of activity along the growth zone but in EU2, tEG1, tAF3, tEG2, and EG4 GPX activity was mainly located in the meristem and in the elongation zone of the leaves (Figure 3H). A subset of lines (EU3, tEU1, AF4, B73, and EG3) closely clustered together showing no spatial differences and very little response to drought (Figure 3H).

## Discussion

Drought is one of the major environmental factors restricting crop production (Boyer, 1982; Al-Kaisi et al., 2013), and a better understanding of the molecular mechanisms involved in the stress response of plants is crucial for selecting plants that are better adapted to these conditions. The response of plants depends on the duration and severity of a drought period, but also on the genetic background and the developmental stage of the plant (Cruz de Carvalho, 2008). To investigate the role of antioxidant regulation in the growth response to stress, we subjected maize lines with different geographical origin and contrasting drought sensitivity to different levels of drought. Using the leaf growth zone as model system, allowed us to study and compare the response of the cells that mediate the growth response (the proliferating and expanding cells) with a high sub-zonal resolution.

Using kinematic analysis to determine the cellular basis of the growth response to drought, we confirmed our previous findings (Avramova et al., 2015a) that the main reason for leaf shortening is a decreased cell division rate, complemented by a small decrease in the length of mature cells. We discovered that different hybrid lines have different strategies to adjust growth under drought conditions. The length and the number of cells in the leaf meristem was most strongly affected in the Egyptian hybrids, cell division rates in the European hybrids and cell elongation rates in the South African hybrids. Despite the reduction in both cell division and cell elongation in response to drought, lines rated as drought tolerant maintain leaf growth due to less compromised meristem sizes and number of dividing cells. This suggests that meristem size control in leaves is a central aspect of drought tolerance in maize. According to the results of the kinematic analysis, the difference in drought tolerance between the Egyptian hybrids was significantly larger than between the European and South-African hybrids. Clearly, the most drought-tolerant lines were tEG1 and tEG2, whereas the most sensitive lines were EG3, EG4, and EG5.

Drought tolerance closely correlated with the MDA concentration in the growth zone (Table S2 and Figure 2A): MDA accumulated particularly in the mature part of the leaves of the sensitive lines in response to the stress showing that these lines experienced more lipid peroxidation. Clearly, the three sensitive EG lines, which experienced the highest reduction in terms of final leaf length in the severe stress conditions, showed the strongest increase in MDA levels in the mature zone of their leaves. The levels of H_2_O_2_ also increased more drastically in the leaves of these three sensitive hybrids compared to the rest of the lines, which explains the higher levels of cellular damage. The patterns of MDA and H_2_O_2_ concentrations group the lines according to drought tolerance, tolerant lines having less impact of the stress (Figures 2A,B) as shown in previous studies (Moussa and Abdel-Aziz, 2008). Consistently, the most sensitive lines (the 3 EG hybrids) clustered together for almost all the measured parameters. They showed a higher induction of their non-enzymatic defense system in response to the drought (total antioxidant capacity, polyphenols, flavonoids). In contrast, the activity of all the redox-regulating enzymes in the severe stress conditions was the least induced in these hybrids (Figure 3). Since these enzyme activities have been related to improved growth during drought and other environmental stressors (Malan et al., 1990; Kraus et al., 1995; Maksimovic et al., 2008; Avramova et al., 2015a), the failure to upregulate their activity in the leaf growth zone could contribute to poor growth of EG3, EG4, and EG5 under drought stress conditions compared to the other lines. In general, the contrast between tEG and EG in terms of growth and redox regulation in response to drought was larger than between tAF and AF and tEU and EU, so that their addition to the panel of hybrids studied earlier (Avramova et al., 2016) was extremely useful.

Changes in the redox status in the leaf growth zone were closely linked to the growth reduction during growth stress. Previously, we showed the changes of the H_2_O_2_ gradient in B73 in response to our drought treatments, and linked these to the mechanisms of leaf growth reduction (Avramova et al., 2015a). Here, we show that the H_2_O_2_ concentration changes in hybrid maize lines follow a pattern similar to B73. However, differences based on the hybrid origin and sensitivity toward drought were clearly visible from the clustering of the lines (Figure 2B). Therefore, even though not being an easy parameter to measure in terms of use for breeding, H_2_O_2_ content is a sensitive parameter to detect responses to drought at the early seedling stage. Moreover, its levels are closely related to the growth response and therefore can discriminate drought tolerance.

The balance of ROS is an integral part of the regulation of meristem size (Tsukagoshi et al., 2010; Avramova et al., 2015a). It is also known that accumulation of H_2_O_2_ negatively impacts the process of cell expansion, enhancing cell wall rigidification (Schopfer, 1996). In our case, the EU hybrids had the smallest reduction of meristem size and cell expansion rate in response to drought stress (Table 1), which could be related to the fact that they had the lowest H_2_O_2_ concentration in both control and stress conditions (Table 3, Figure 2B). The hybrids with the highest amount of H_2_O_2_ in both division and elongation zones, during drought stress, EG3, EG4, and EG5, also experienced the highest reduction in cell division and elongation rates. On the other hand, the tEG hybrids had slightly higher H_2_O_2_ levels than the rest of the tolerant hybrids in their elongation zone in the stress conditions but had a smaller reduction in cell elongation rates. This could be related to the fact that, next to the damaging effect of H_2_O_2_ accumulation in the leaf, basal H_2_O_2_ concentrations promote cell elongation by mediating cell wall loosening shown in roots (Liszkay et al., 2004; Tsukagoshi et al., 2010), but also in maize leaves (Rodriguez et al., 2002; Shoresh et al., 2011). During salinity stress, leaf and root cells demonstrate different requirements for ROS in order to regulate their growth (Bernstein et al., 2010) and in maize leaves ROS scavenging mechanisms are coupled with increased cell-wall rigidity and therefore reduced cell expansion (Kravchik and Bernstein, 2013). Therefore, our results are in agreement with the hypothesis that ROS have and important function as growth-regulating signals and depending on their concentration they can both promote or restrict organ growth (Tsukagoshi et al., 2010; Schmidt et al., 2016), which is related to the important role of H_2_O_2_ as a cellular messenger during the stress response triggering cell-signaling and gene-expression patterns (Schieber and Chandel, 2014).

The kinematic analysis pointed at significant differences in the growth of the hybrids from different geographic origins, the Egyptian hybrids (tEG and EG) being most different from the others. Our biochemical measurements showed a clear separation between the Egyptian and other hybrids according to most of the measured parameters (ASC, GSH, tASC, tGSH, total antioxidant capacity, polyphenols, flavonoids, SOD, CAT, POX, DHAR, MDAR). This could be due to the fact that, unlike the African and the European hybrids, which were all provided by DuPont Pioneer, the Egyptian lines originate from a different breeding program and have a different genetic background (Sids Research Station, Agricultural Research Centre, Beni-Suef, Egypt).

The inbred line B73 responded differently to the stress than the hybrids in terms of its growth (*LL*, Table 1) and also in terms of the H_2_O_2_ concentrations and the activities of CAT, POX, and APX in its leaves (Table 2). According to the patterns of distribution along the leaf axis, B73 clustered separately from the hybrids in terms of H_2_O_2_, total antioxidant capacity, ASC, and CAT. Based on these findings we can conclude that the differences in shoot, root, and leaf growth parameters between hybrids and this inbred line (Avramova et al., 2016), are associated with molecular level changes in redox regulation. Previously, we demonstrated the involvement of redox regulation in maintaining growth under drought conditions (Avramova et al., 2015a), and the lower CAT, POX, and APX activities in the leaves of B73 provide an explanation for the higher levels of H_2_O_2_ and consequently to its reduced growth under drought stress compared to the hybrid lines.

An interesting finding of the current study is that the drought tolerance is not mediated by the same responses in the hybrids, provided by DuPont Pioneer (West European and South African) and the Egyptian hybrids. Our PCA analysis (Figure 1) clearly shows that the Egyptian hybrids respond at the metabolite level and the rest of the hybrids at the enzyme level. These findings point out that plants have developed different strategies to respond to drought, not only at the cellular (demonstrated by our kinematic analysis), but also at the molecular level. Nevertheless, the most drought tolerant tEG hybrids showed higher levels of both metabolite concentrations and enzyme activities, demonstrating a combined strategy as the most successful to maintain leaf growth in water deficit conditions.

The differences in the response to the drought conditions between hybrids with different drought tolerance in the field (based on breeder's ranking), different origin and between hybrids and the inbred line at the early seedling stage, allowed us to address the question which parameters are the most useful to detect drought tolerance. According to the ANOVA analysis (Table 2), with the exception of GPX, the treatment effect was significant for all measured parameters. Most of the parameters also showed significant differences for the zone effect, implying consistent patterns of concentrations (or activities for the enzymes) along the developmental gradient of the leaf across the lines (except for GPX, DHAR, polyphenols, and flavonoids). The response of the lines from different origin (EU, AF, and EG) was significantly different according all the measured parameters, while differences in drought tolerance were significant for most of them, except for GPX, SOD, H_2_O_2_, and tASC. The interaction effects D*O, O*T, and D*O*T showed that the responses of the tolerant lines from different origins differed according to most of the parameters. Clearly, the enzyme activities (especially those of GR, CAT, and APX) had higher number of significant factors and between-factors interactions than the metabolite concentrations, suggesting that they are more useful parameters to identify differences in drought tolerance between lines.

In conclusion, our results show that variations in drought tolerance are detectable at the early seedling stage and can be explained by different redox regulation in the growth zone of the leaves. Moreover, all the measured parameters such as ROS, stress determinants (MDA), antioxidant molecules and redox enzyme activities distinguish the geographical origins of the lines. The results indicate that there are different strategies of coping with the stress at the cellular level that at the molecular level relate to the regulation of ROS levels in the leaf growth zone. According to our results, tolerant maize hybrids experience a smaller impact of drought on cell division due to a smaller reduction of leaf meristem size and number of dividing cells. The leaf meristems of these hybrids are better protected during the stress, particularly due to a higher activity of the redox-regulating enzymes CAT, POX, APX, and GR, resulting in less H_2_O_2_ production in these zones, allowing improved growth under drought conditions.

## Author contributions

VA and HAb designed and performed the experiments, compiled the data and wrote the article; AP, IV, AH, and JM performed the experiments; HAs supervised the experiments; GB conceived the project; all authors contributed to the writing.

## Funding

This work was supported by grants from the Interuniversity Attraction Poles Program (Belgian Network MARS: “Growth and Development of Higher Plants”; IUAP VII/29), of the Science Policy Office of the Belgian State, Research grant G0D0514N from the Flemish Science Foundation, a PhD fellowship of the University of Antwerp to VA, and Erasmus grants to AP, AH, and IV. The funders had no role in study design, data collection and analysis, decision to publish, or preparation of the manuscript.

### Conflict of interest statement

The authors declare that the research was conducted in the absence of any commercial or financial relationships that could be construed as a potential conflict of interest.
